# The Adhesion G Protein-Coupled Receptor GPR97/*ADGRG3* Is Expressed in Human Granulocytes and Triggers Antimicrobial Effector Functions

**DOI:** 10.3389/fimmu.2018.02830

**Published:** 2018-12-03

**Authors:** Cheng-Chih Hsiao, Tai-Ying Chu, Chia-Jung Wu, Maartje van den Biggelaar, Caroline Pabst, Josée Hébert, Taco W. Kuijpers, Brendon P. Scicluna, Kuan-Yu I, Tse-Ching Chen, Ines Liebscher, Jörg Hamann, Hsi-Hsien Lin

**Affiliations:** ^1^Department of Experimental Immunology, Amsterdam UMC, University of Amsterdam, Amsterdam, Netherlands; ^2^Department of Microbiology and Immunology, College of Medicine, Chang Gung University, Taoyuan, Taiwan; ^3^Sanquin Research and Landsteiner Laboratory, Department of Plasma Proteins, Amsterdam UMC, University of Amsterdam, Amsterdam, Netherlands; ^4^Department of Internal Medicine V, Heidelberg University, Heidelberg, Germany; ^5^Division of Hematology-Oncology and Leukemia Cell Bank of Quebec, Maisonneuve-Rosemont Hospital, Montreal, QC, Canada; ^6^Department of Medicine, University of Montreal, Montreal, QC, Canada; ^7^Sanquin Research and Landsteiner Laboratory, Department of Blood Cell Research, Amsterdam UMC, University of Amsterdam, Amsterdam, Netherlands; ^8^Department of Pediatric Hematology, Immunology and Infectious Diseases, Emma Children's Hospital, Amsterdam UMC, Amsterdam, Netherlands; ^9^Department of Clinical Epidemiology, Amsterdam UMC, University of Amsterdam, Amsterdam, Netherlands; ^10^Center for Experimental Molecular Medicine, Amsterdam UMC, University of Amsterdam, Amsterdam, Netherlands; ^11^Department of Anatomic Pathology, Chang Gung Memorial Hospital-Linkou, Taoyuan, Taiwan; ^12^Rudolf Schönheimer Institute of Biochemistry, Medical Faculty, Leipzig University, Leipzig, Germany; ^13^Chang Gung Immunology Consortium, Chang Gung Memorial Hospital-Linkou, Taoyuan, Taiwan

**Keywords:** adhesion GPCR, monoclonal antibody, granulocytes, inflammation, G-protein signaling, antimicrobial activity

## Abstract

The adhesion family of G protein-coupled receptors (aGPCRs) comprises 33 members in human, several of which are distinctly expressed and functionally involved in polymorphonuclear cells (PMNs). As former work indicated the possible presence of the aGPCR GPR97 in granulocytes, we studied its cellular distribution, molecular structure, signal transduction, and biological function in PMNs. RNA sequencing and mass-spectrometry revealed abundant RNA and protein expression of ADGRG3/GPR97 in granulocyte precursors and terminally differentiated neutrophilic, eosinophilic, and basophilic granulocytes. Using a newly generated GPR97-specific monoclonal antibody, we confirmed that endogenous GPR97 is a proteolytically processed, dichotomous, N-glycosylated receptor. GPR97 was detected in tissue-infiltrating PMNs and upregulated during systemic inflammation. Antibody ligation of GPR97 increased neutrophil reactive oxygen species production and proteolytic enzyme activity, which is accompanied by an increase in mitogen-activated protein kinases and IκBα phosphorylation. In-depth analysis of the GPR97 signaling cascade revealed a possible switch from basal Gαs/cAMP-mediated signal transduction to a Gαi-induced reduction in cAMP levels upon mutation-induced activation of the receptor, in combination with an increase in downstream effectors of Gβγ, such as SRE and NF-κB. Finally, ligation of GPR97 increased the bacteria uptake and killing activity of neutrophils. We conclude that the specific presence of GPR97 regulates antimicrobial activity in human granulocytes.

## Introduction

Polymorphonuclear cells (PMNs), including neutrophilic, eosinophilic, and basophilic granulocytes, execute highly effective responses against microorganisms, which are of critical importance for tissue homeostasis and wound healing (1). Key to these activities, performed in concert with other immune cells, is the ability of PMNs to respond to environmental signals through cell surface proteins, including Toll-like receptors, C-type lectin-like receptors, Fc receptors, anaphylatoxin receptors, cytokine receptors, and G protein-coupled receptors (GPCRs) (2). Next to classical GPCRs with their established functions in chemotaxis and complement binding, PMNs express members of the adhesion GPCR (aGPCR) family (3, 4). aGPCRs contain a large extracellular domain (ECD) with various protein folds that, through a GPCR autoproteolysis-inducing (GAIN) domain, is linked to the seven-span transmembrane (7TM) domain and the cytoplasmic tail (5). The GAIN domain facilitates self-catalyzed proteolytic processing of many aGPCRs at a juxtamembranous GPCR proteolysis site (GPS) into an extracellular N-terminal fragment (NTF) and a 7TM/cytoplasmic C-terminal fragment (CTF) (6–8). The cleaved NTF and CTF of aGPCRs normally remain non-covalently associated at the cell surface, hence permitting the dichotomous NTF–CTF receptor complex a dual role in cell adhesion and signaling (5, 9).

We previously reported expression of the EGF-TM7/*ADGRE* subfamily aGPCRs EMR1/*ADGRE1*, EMR2/*ADGRE2*, EMR3/*ADGRE3*, and CD97/*ADGRE5* in PMNs (10–14). Ligation of EMR2 regulates human neutrophil function, including adhesion, migration, reactive oxygen species (ROS) production, and proteolytic enzyme degranulation (15). More recently, expression of another gene cluster, encoding the *ADGRG* subfamily members GPR56/*ADGRG1*, GPR97/*ADGRG3*, and GPR114/*ADGRG5*, was found in immune cells (16). While GPR56 is specifically expressed in human cytotoxic lymphocytes, where it inhibits immediate effector functions (17), the cellular distribution, and molecular function of GPR97 and GPR114 remained poorly understood (3, 4). Earlier studies in mice have delineated a role of GPR97 in lymphoid development, in particular B-lymphocyte fate decision (18, 19). *Adgrg3* is repressed by the transcription factor Pax5 (20) but activated by the Pax5-Etv6 oncoprotein in B-cell acute lymphoblastic leukemia (21). In addition, more infiltrating macrophages and higher tumor necrosis factor levels were identified in the liver and kidney of *Adgrg3*-deficient mice, compared to wild-type animals, in a high-fat diet-induced experimental obesity model (22). By contrast, GPR97 was shown to be insignificant for inflammation in an ovalbumin-induced asthma model (23). Outside the immune system, GPR97 regulates adhesion, and migration of lymphatic endothelial cells through the modulation of RhoA and Cdc42 activity (24).

We here present transcriptomic and proteomic data that demonstrate the presence of GPR97 in all granulocyte lineages and reveal its upregulation during systemic inflammation. Using a newly generated monoclonal antibody (mAb) directed against the NTF, we confirm a dichotomous structure of GPR97, show its expression in tissue-infiltrating PMNs, and provide evidence that crosslinking of the receptor promotes the production of antimicrobial mediators by PMNs. We further indicate the possibility that a switch in Gα signaling upon GPR97 activation causes a reduction in cAMP levels in combination with an increase in downstream effectors of Gβγ. We conclude that GPR97 is a specific aGPCR and functional modulator of human granulocytic leukocytes.

## Materials and methods

### Analysis of transcriptomic and proteomic datasets

Genome-wide gene expression data of sorted human cord blood, bone marrow, and peripheral blood cell populations, including CD34^−^CD11b^−^CD16^−^ promyelocytes, CD34^−^CD11b^+^CD16^−^ myelocytes, CD34^−^CD11b^+^CD16^med^ metamyelocytes, CD34^−^CD11b^+^CD16^+^ band cells/segmented granulocytes, SSC^high^CD33^+^ granulocytes, FSC^high^SSC^low^CD33^++^CD14^+^ monocytes, SSC^low^CD3^+^ T cells, CD34^+^CD10^+^CD19^+^ pre-B cells-I, SSC^low^CD19^+^ pre-B cells-II, and CD34^−^CD10^+^CD19^+^ B cells (GSE48846, GSE98310, GSE51984) were derived from (25).

Cellular proteomics data have been obtained by liquid chromatography–mass spectrometry (LC-MS)/MS and MaxQuant's label-free quantification (MaxLFQ). Data of human granulocytic leukocytes, including neutrophils, eosinophils, and basophils (PXD004352), were derived from Rieckmann et al. (26). Data of flow cytometry-sorted myeloid progenitor cells derived from bone marrow, i.e., (pro)myelocytes, metamyelocytes, immature neutrophils with a band-formed nucleus, and mature neutrophils with segmented nuclei, and from blood (PMNs) were derived from van den Biggelaar et al. (manuscript in preparation). Linked genome-wide gene expression data of the same cell populations were derived from Grassi et al. (27).

Genome-wide gene expression analysis of PAXgene whole blood from critically ill patients with community-acquired pneumonia was done by hybridization to Affymetrix U219 96-array chips (Affymetrix, Santa Clara, CA, USA; GSE65682) (28, 29), whereas whole blood from a human endotoxemia cohort (4 ng *Escherichia coli* lipopolysaccharide (LPS) per kg body weight) was analyzed using the Illumina HumanHT-12 V3.0 expression beadchip (Illumina; GSE48119) (30, 31). Comparison of community-acquired pneumonia patients relative to healthy subjects as well as post-LPS administration (4 h) to pre-LPS administration were done by moderated *t*-tests, implemented in the Limma method (32).

### Generation of a GPR97-specific mAb

Human GPR97-ECD–mFc (fragment crystallizable) fusion protein was produced by transfecting a pSecTag2A-mFc vector (Invitrogen, San Diego, CA, USA), containing the entire ECD of GPR97, into HEK-293T cells, and purifying the fusion protein on a Protein A Sepharose™ 4 Fast Flow column (GE Healthcare, Little Chalfont, United Kingdom) (33). Protein was injected subcutaneously into BALB/c mice to generate GPR97-specific mAb. In brief, 100 μg GPR97-ECD–mFc with complete Freund's adjuvant was injected into mice for the first antigen (Ag) challenge. Immunized mice were boosted with 100 μg Ag with incomplete adjuvant at days 21, 35, and 51. A final boost of 50 μg Ag without adjuvant was given to mice at day 65. Splenocytes from sensitized mice were purified and fused with NS-1 myeloma cells to generate hybridoma cells, which were selected and subcloned in hypoxanthine–aminopterin–thymidine medium to identify a GPR97-specific clone by enzyme-linked immunosorbent assay (ELISA) as described previously (16), using GPR97-ECD–mFc as Ag and purified mFc fragment as a negative control.

### Isolation of human PMNs

PMNs were separated from fresh blood collected in tubes containing sodium heparin and isolated by Polymorphprep^TM^ density gradient centrifugation (Axis-Shield, Oslo, Norway). All procedures were approved by the Chang Gung Memorial Hospital Ethics Committee (CGMH IRB No: 201601053B0) and performed according to the guideline set by the Committee. PMNs of >98% purity, yielded by hypotonic lysis of erythrocytes of the PMN-enriched fraction, were resuspended in Hank's balanced salt solution (HBSS)/0.1% bovine serum albumin (BSA) for subsequent use.

### Western blot analysis

To obtain total cell lysates for Western blot analysis, harvested cells were spun down at 1,500 rpm for 5 min at 4°C, washed once with ice-cold 1 × HBSS, and lysed in 100 μl ice-cold modified cell lysis buffer as described previously (17). The bicinchoninic acid protein assay kit (Pierce, Rockford, IL, USA) was used to quantify protein samples, which were then subjected to sodium dodecyl sulfate (SDS)-polyacrylamide gel electrophoresis and transferred to polyvinylidene fluoride membranes (Millipore, Bedford, MA, USA). Blotted membranes were incubated in blocking buffer [5% of BSA in phosphate-buffered saline (PBS)] for 1 h, followed by 1 h incubation with the first Ab in blocking buffer at RT. Following extensive washes in blocking buffer, membranes were incubated with horseradish peroxidase (HRP)-conjugated second Ab (Sigma-Aldrich, St. Louis, MO, USA) in blocking buffer. When indicated, HRP-conjugated anti-mouse IgG (Fab-specific; Sigma-Aldrich). Membranes were extensively washed, and the bound HRP signal was detected by chemiluminescence (Amersham ECL, GE Healthcare, or SuperSignal West Pico PLUS, Pierce) for 5 min. A mAb against GPR56 (clone CG4) was produced in house (16). (m)Abs against ERK1/2 (clone 137F5), phospho-ERK1/2 (Thr202/Tyr204; #4370), p38 (#9212), phospho-p38 (Thr180/Tyr182; #9211), IκBα (clone 44D4), phospho-IκBα (Ser32; clone 14D4), and Myc (clone R950-25) were obtained from Cell Signaling Technology (Beverly, MA, USA) and Invitrogen, respectively.

### Immunohistochemical staining

*In situ* expression patterns of GPR97 were studied using standard immunohistochemistry by the fully automated Leica Bond Max slide stainer (Leica Biosystems, Wetzlar, Germany). Briefly, paraffin wax blocks of human tissue samples were sectioned (~4–5 μm) and fixed on slides. Ag retrieval was performed on the tissue sections, which were then incubated with the 2 μg/ml G97-A mAb in blocking buffer (5% of BSA in PBS) at 4°C for 1 h. After extensive washes, tissue sections were incubated with the standard Bond-max reagents as previously described (34). Staining was revealed following the addition of appropriate substrates and observed by microscope.

### Generation of full-length and mutant GPR97 constructs

A full-length mouse *Adgrg3* sequence was amplified from a lung cDNA library and directly cloned into the mammalian expression vector pcDps (forward primer: 5′-gcaggaagaaggtcagttgg-3′; reverse primer: 5′-agaagacagtggagcccaga-3′). For detection purpose and to increase cell surface expression, a hemagglutinin (HA) epitope was inserted directly downstream the predicted signal peptide (SignalP 4.1 server; http://www.cbs.dtu.dk/services/SignalP) by a PCR-based site-directed mutagenesis and fragment replacement strategy. Further, a Flag epitope was introduced at the very C-terminus.

A GPR97 mutant construct, replacing the natural N-terminal fragment (amino acids 1–244) with the N-terminus of P2Y_12_ (amino acids 1–34, including the N-terminal HA tag at amino acid positions 2–10), referred to as the CTF-only mutant, was generated by PCR and homologous recombination in *E. coli* (Invitrogen). Fidelity of full-length and CTF-only mutant GPR97 constructs was verified by sequencing.

### *In vitro* signal transduction assays

GPR97 constructs were heterologously expressed in COS-7 cells, grown in Dulbecco's minimum essential medium (DMEM), supplemented with 10% fetal bovine serum (FBS), 100 units/ml penicillin, and 100 μg/ml streptomycin at 37°C and 5% CO_2_ in a humidified atmosphere. For cyclic adenosine monophosphate (cAMP) accumulation assays, cells were split into 48-well plates at a density of 3 × 10^4^ cells/well and transfected with 600 ng of plasmid DNA/well using Lipofectamine^TM^ 2000 (Invitrogen) according to the manufacturer's protocol. Forty-eight hours after transfection, cells were incubated with 3-isobutyl–methyl–xanthine (1 mM; Sigma-Aldrich)-containing medium. Parallel stimulation with forskolin (10 μM; Sigma-Aldrich) served as positive control. Cells were lysed in LI buffer (PerkinElmer, Rodgau, Germany) and kept frozen at−20°C until measurement. To measure cAMP concentration, the Alpha Screen cAMP assay kit (PerkinElmer) was used according to the manufacturer's protocol. The accumulated cAMP was measured in 384-well white OptiPlate microplates (PerkinElmer) with the Fusion AlphaScreen multilabel reader (PerkinElmer).

For luciferase reporter gene assays, HEK-293T cells were grown in DMEM, supplemented with 10 % FBS, 100 units/ml penicillin, and 100 μg/ml streptomycin at 37°C in a humidified 5% CO_2_ incubator. One day prior to transfection, cells were split into 96-well cell culture plates (3 × 10^4^ cells/well), and 24 h later, cells were co-transfected with the empty vector control or the given receptor expression plasmid (100 ng/well each) and the indicated Luciferase reporter plasmid (50 ng/well, PathDetect Reporting System; Agilent, Santa Clara, CA, USA). Lipofectamine^TM^ 2,000 (Invitrogen) was used for transient transfection. After transfection, cells were maintained in antibiotic-free medium (10% serum, except for serum response element (SRE) and serum response factor (SRF) measurements) throughout the experiments. Twenty nine hours after transfection, the assay was terminated by washing the cells twice with PBS and addition of 100 μl luciferase assay reagent (SteadyLite; PerkinElmer), and fluorescence was measured with the EnVision Multilabel plate reader (PerkinElmer).

To estimate cell surface expression of receptors carrying an N-terminal HA tag, an indirect cellular ELISA was used (35). To assess the amounts of full-length HA/Flag double-tagged constructs in the cell, a sandwich ELISA was performed (36).

For analysis of the activation of signaling molecules, freshly isolated PMNs were washed in HBSS and re-suspended in serum-free RPMI medium. Cells (4 × 10^6^ cells/ml/tube) were placed in Eppendorf tubes pre-coated with 0.1 % BSA. Cells were incubated with or without G97-A mAb (10 μg/ml) for 30 min at 4°C, followed by the addition of goat anti-mouse IgG F(ab')2 (#115-006-062, 10 μg/ml; Jackson ImmunoResearch, West Grove, PA, USA) for 30 min at 4°C to allow receptor crosslinking. Tubes were then transferred to 37°C water bath and stopped at indicated time points (5, 10, 15 min) by placing tubes at 4°C ice bucket. Cell lysate was collected, quantified, and analyzed by Western blotting using appropriate mAbs. When necessary, cells were pre-treated with specific signaling inhibitors as indicated. In addition, PMNs were treated with control mouse IgG1 (clone 11711, 10 μg/ml; R&D Systems, Minneapolis, MN, USA) and LPS (100 ng/ml; Sigma-Aldrich) as a negative and positive control, respectively.

### Analysis of ROS production and MPO activity

Freshly isolated human PMNs (2 × 10^6^ cells/ml) were resuspended in PBS, supplemented with 0.2% BSA and 5 mM glucose, and incubated with dihydrorhodamine-123 (2 μM; Invitrogen) for 30 min at RT. Cells were then incubated with control IgG1 or with soluble or immobilized G97-A mAb (1, 2, 5, 10, and 20 μg/ml) for 30 min at 37°C in the absence or presence of N-formyl–Met–Leu–Phe (fMLF, 1 μM; Sigma-Aldrich), before being placed on ice to stop the reaction. The accumulation of H_2_O_2_ was measured immediately by flow cytometry on a FACSCalibur machine (BD Biosciences, San Diego, CA, USA), and data were analyzed with FlowJo software version 7.6.1 (Tree Star, Ashland, OR, USA). To measure myeloperoxidase (MPO) activity, cell lysate was collected and analyzed using the Myeloperoxidase Activity Colorimetric Assay Kit (BioVision, Milpitas, CA, USA) according to the manufacturer's recommendations.

For signaling inhibitor treatment, PMNs were pre-incubated with the indicated reagents at 37°C for 30 min: PD98059 (20 μM; Cayman Chemical, Ann Arbor, MI, USA), U0126 (10 μM; Promega, Madison, WI, USA), Bay 11–7082 (5 μM; InvivoGen, San Diego, CA, USA), SP600125 (20 μM; Sigma-Aldrich), SB203580 (20 μM; Cayman Chemical) or N-acetylcysteine (NAC, 10 mM; Sigma-Aldrich) before proceeding with the functional assay.

### Phagocytosis and bacteria killing assays

Freshly isolated neutrophils (1 × 10^5^ cells/well) were suspended in 100 μl RPMI in 96-well plates and incubated with immobilized control IgG1 or G97-A mAb (10 μg/ml) for 30 min at 37°C. To measure bacteria uptake, 10 μl pHrodo Green *E. coli* BioParticles (Invitrogen) were added to the cells for 1 h. When indicated, cells were treated with fMLF (1 μM) or cytochalasin D (10 μg/ml) as positive and inhibition control, respectively. Bacteria phagocytosis was immediately quantified by flow cytometry. For the uptake of live *E. coli*, freshly isolated neutrophils (5 × 10^5^ cells/well) were suspended in 400 μl RPMI in 24-well plates and incubated with indicated concentrations of immobilized control IgG1 or G97-A mAb for 30 min at 37°C. A late logarithmic-phase grown *E. coli* (strain DH10B, multiplicity of infection (MOI) 100) was added for 1 h at 37°C. Extracellular bacteria were removed, and cells were washed by PBS and incubated for 30 min in RPMI medium containing 50 μg/ml gentamicin. Subsequently, PMNs were washed twice with PBS and lysed with 0.05% Triton X-100 in PBS. Several dilutions of the lysate were plated on LB plates and incubated overnight at 37°C. *E. coli* colony-forming units (CFUs) were counted the following day.

To analyze bacteria killing, freshly isolated PMNs (1.25 × 10^6^ cells/ml) were incubated with *E. coli* (strain DH10B, MOI 100) at 37°C for 1 h to allow bacteria phagocytosis. After extensive washing to remove extracellular bacteria and incubation in RPMI medium containing gentamicin, PMNs were suspended in 100 μl RPMI and incubated with indicated concentrations of immobilized control IgG1 or G97-A mAb at 37°C. At chosen time points, cells were lysed with 0.05% Triton X-100 in PBS. Several dilutions of the lysate were plated on LB plates and incubated overnight at 37°C. The following day, *E. coli* CFUs were counted.

### Statistical analysis

All quantitative data were analyzed using GraphPad Prism (version 6.0; GraphPad Software, San Diego, CA, USA) and expressed as means ± standard error of the mean (SEM) with the number of experimental replicates (n) provided. Differences between groups were determined by Student's *t*-test. In all cases, a probability value of < 0.05 was accepted to reject the null hypothesis. The statistical significance of *p* was set at ^*^p < 0.05, ^**^p < 0.01, and ^***^p < 0.001.

## Results

### Expression of GPR97 by human granulocytes

**Figure 1:**
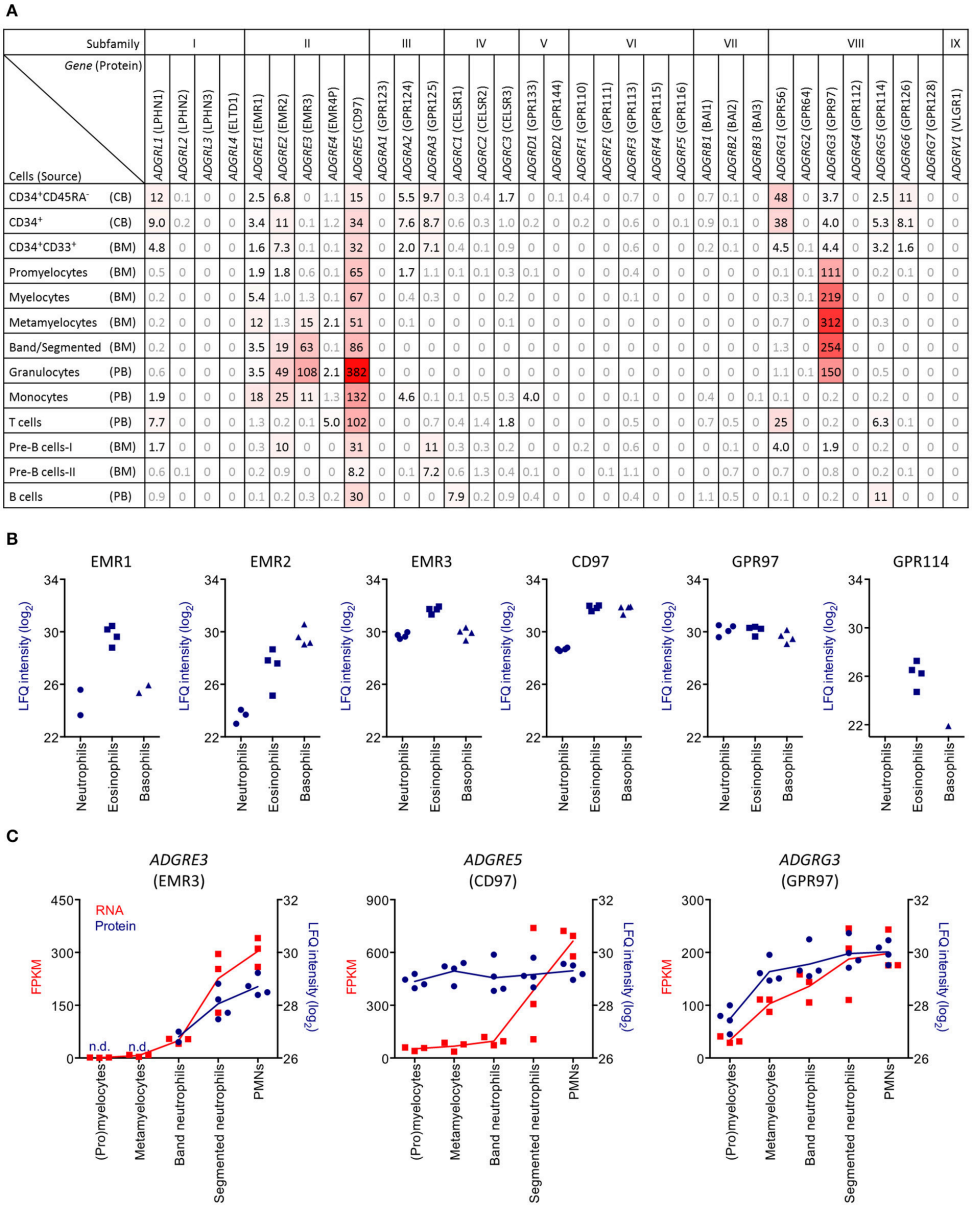
PMNs specifically express *ADGRG3* (GPR97). **(A)** RNA sequencing data showing aGPCR expression in sorted cord blood (CB), bone marrow (BM), and peripheral blood (PB) cell populations, derived from Maiga et al. (25). Shown are aGPCR with reads per kilobase per million mapped reads (RPKM) >1.5. **(B)** Mass spectrometry data showing protein expression of the aGPCRs EMR1, EMR2, EMR3, CD97, GPR97, and GPR114 in granulocyte populations, including neutrophils, eosinophils, and basophils, derived from Rieckmann et al. (26). **(C)** Linked RNA sequencing and mass spectrometry data showing expression of the aGPCRs *ADGRE3* (EMR3), *ADGRE5* (CD97), and *ADGRG3* (GPR97) in precursor and mature granulocytes. FPKM, fragments per kilobase per million mapped reads; LFQ, label-free quantification.

Our previous microarray study indicated mRNA expression of *ADGRG3* (GPR97) in human PMNs (16). By analyzing publicly available RNA-sequencing data from sorted human bone marrow, cord blood, and peripheral blood cells, we revisited this finding and obtained detailed information about the expression of aGPCRs during hematopoietic differentiation (Figure 1A). We found that eleven of the 33 human aGPCR genes are potentially expressed in hematopoietic stem and precursor cells (HSPCs) from cord blood and bone marrow. Expression in mature hematopoietic cells was restricted to six aGPCRs. The most widely expressed receptor, found in all hematopoietic lineages, was *ADGRE5* (CD97). In contrast, *ADGRG1* (GPR56) was exclusively expressed in HSPCs and in cytotoxic lymphocytes, while expression of *ADGRE1, ADGRE2*, and *ADGRE3* (EMR1, EMR2, and EMR3) was fairly restricted to (precursor) myeloid cells. Notably, *ADGRG3* was very abundantly transcribed during the developmental stages of PMNs, ranging from promyelocytes to mature granulocytes.

We verified aGPCR gene expression at the protein level using a high-resolution MS-based proteomics data set of 28 primary human hematopoietic cell populations generated by Rieckmann and colleagues (26). We found six aGPCRs to be expressed in PMNs (Figure 1B). As expected, EMR1, the human homolog of F4/80, was restricted to eosinophils (10, 37). Further confirming previous findings, EMR2, EMR3, and CD97 were found in all granulocytic lineages, although surface expression in neutrophilic granulocytes was absent/low in resting cells that upregulate surface expression during cellular activation (11, 12, 15, 38). GPR97 was detected at comparable levels in neutrophilic, eosinophilic, and basophilic granulocytes. Finally, GPR114 was found in eosinophils, which is in line with the previously reported transcription of the encoding gene *ADGRG5* in these cells (16).

We next directly compared mRNA and protein expression of aGPCRs during neutrophilic granulocyte development (Figure 1C). We confirmed a late upregulation of EMR3 protein during granulocyte maturation, which matches the transcription kinetics and confirms the molecule to be a marker for PMN maturation (12). In contrast, CD97 protein was highly expressed during all stages of granulocyte development, which possibly is due to its steady, albeit lower, mRNA expression in HSPCs. Finally, *ADGRG3* (GPR97) transcription and translation were closely correlated and induced early during metamyelocyte formation. In conclusion, human granulocytes but no other immune cells broadly express GPR97.

### GPR97 is an N-Glycosylated bipartite receptor

**Figure 2:**
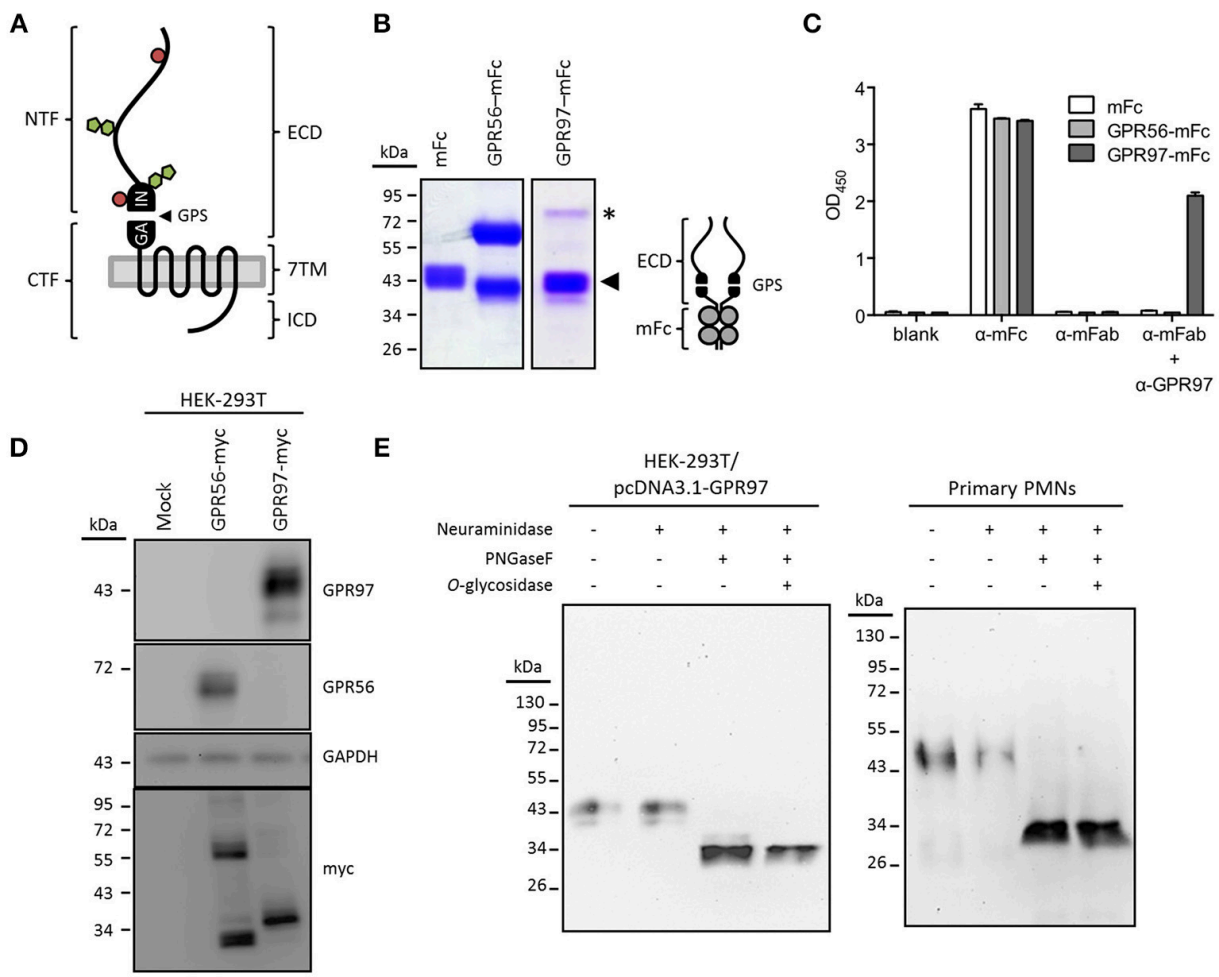
GPR97 is a dichotomous receptor**. (A)** Schematic presentation of GPR97 architecture with the N-terminal and C-terminal fragment (NTF and CTF), linked by the (GPCR autoproteolysis-inducing) GAIN domain with a GPCR proteolysis site (GPS), predicted to facilitate autoproteolysis of the receptor. Alternatively, the protein layout of GPR97 is marked by a three-partite structure consisting of an extracellular domain (ECD), a seven transmembrane (7TM) domain, and an intracellular domain (ICD). N- and O-glycosylation sites were predicted by NetNGlyc and NetOGlyc (http://www.cbs.dtu.dk/services/) and are indicated by green pentagons and red circles, respectively. **(B)** SDS-PAGE analysis of the GPR97-ECD–mFc protein (schematically provided to the right) used for mAb production. The molecular weight of GPR97-NTF is ~43 kDa (indicated by arrowhead), similar to the size of mFc. A minor fraction of uncleaved GPR97-ECD–mFc was expressed as a ~80-kDa band (indicated by asterisk). mFc and GPR56-ECD–mFc protein were included as controls. **(C)** ELISA demonstrating the specificity of the anti-GPR97 mAb G97-A. ELISA plates coated with mFc, GPR56-ECD–mFc, or GPR97-ECD–mFc protein were probed with Abs directed against mouse IgG Fc, mouse IgG Fab, or GPR97 (G97-A), followed by color development of tetramethylbenzidine substrate. **(D)** Western blot analysis of GPR97 receptor expressed in HEK-293T cells transiently expressing GPR97–myc. Data show that GPR97 is proteolytically cleaved into a ~43-kDa NTF and a ~38-kDa CTF, detected by anti-GPR97 (G97-A) and anti-myc mAbs, respectively. HEK-293T cells expressing GPR56–myc protein were included as controls. **(E)** Total cell lysates of HEK-293T cells transiently expressing GPR97 and primary PMNs were treated with or without neuraminidase, PNGaseF, and *O*-glycosidase and probed with G97-A mAb. As indicated, GPR97 protein was heavily decorated by N-linked glycosylation.

GPR97 contains an O- and N-glycosylated ECD of ~250 amino acids that comprises the GAIN domain (Figure 2A). To start with the molecular characterization of GPR97 protein in human granulocytes, we generated a GPR97-specific mAb, designated G97-A, in mice using a GPR97-ECD–mFc fusion protein as Ag (Figure 2B). ELISA and Western blot analyses showed that G97-A mAb reacted specifically with the GPR97-ECD and recognized a specific 43-kDa fragment in the lysate of HEK-293T cells expressing a full-length GPR97 with a C-terminally tagged myc-epitope, but not mocked transfected cells or those expressing GPR56-myc (Figures 2C,D). Importantly, a distinct ~38-kDa fragment was detected when the same blot was probed with a myc mAb to identify the myc-tagged 7TM fragment (Figure 2D; Figure S1

In line with the data obtained from transiently transfected HEK-293T cells, a ~43-kDa band of endogenous GPR97 was detected in protein lysates of human primary PMNs (Figure 2E) but not monocytes or lymphocyte (data not shown). Finally, deglycosylation showed that both, exogenously and endogenously expressed GPR97 receptors, are heavily N-glycosylated proteins with a core molecular weight of ~34 kDa (Figure 2E). These results confirmed that GPR97 is a glycosylated cell surface protein, proteolytically processed at the GPS motif, similar to GPR56 and various other aGPCRs.

### Tissue-infiltrating granulocytes express GPR97

**Figure 3:**
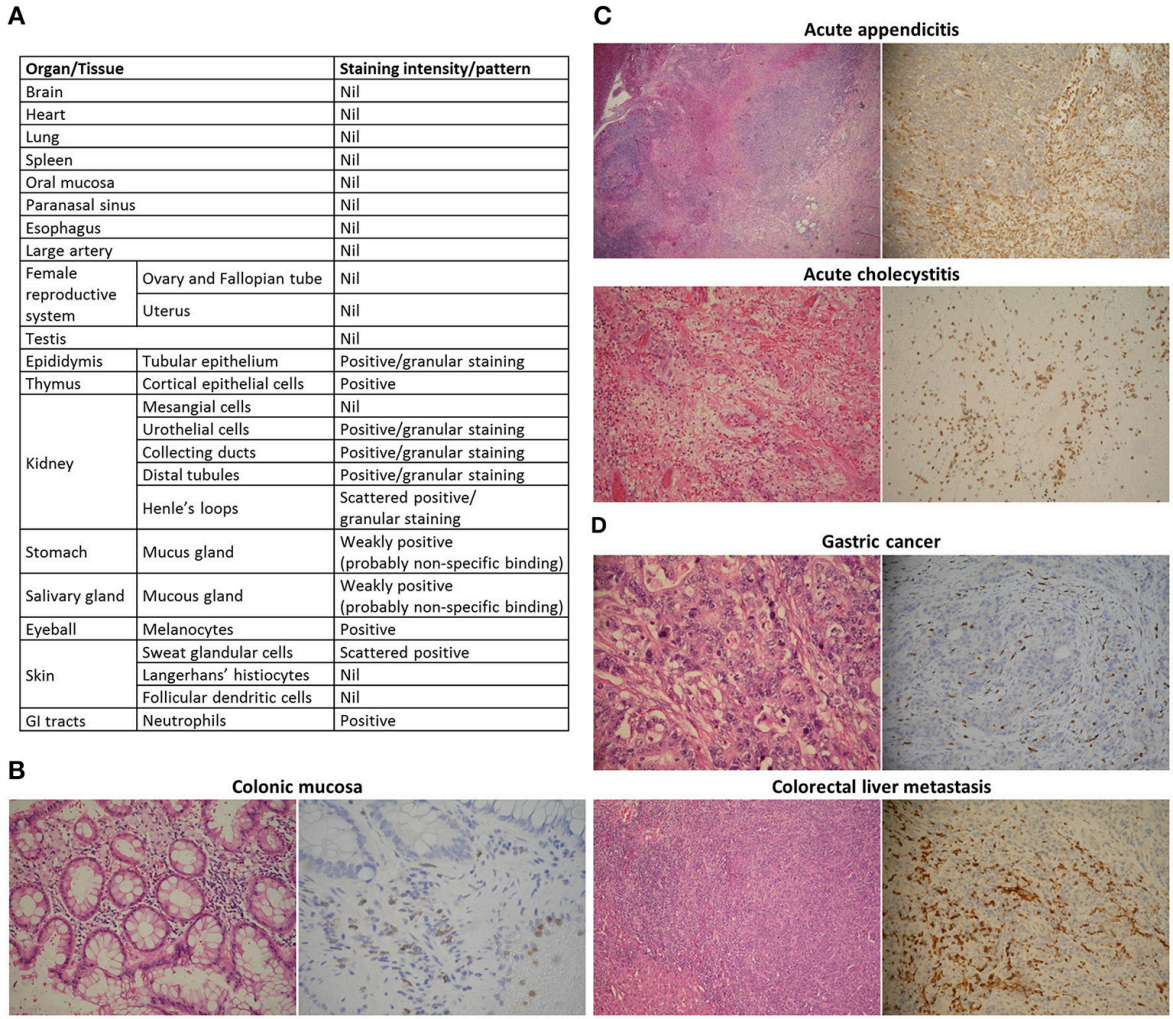
Tissue-infiltrating PMNs express GPR97. Tissue reactivity of the anti-GPR97 mAb G97-A on formalin-fixed and paraffin-embedded human tissues. **(A)** Summary of GPR97 expression in human organs. **(B**–**D)** Left: hematoxylin and eosin staining. Right: immunohistochemical staining. G97-A-stained neutrophils in **(B)** colonic mucosa (400 × ), **(C)** acute appendicitis and acute cholecystitis (both 200 × ), and **(D)** gastric adenocarcinoma and colorectal liver metastasis (both 200 × ).

We then asked whether the G97-A mAb is able to recognize neutrophils in formalin-fixed and paraffin-embedded tissue sections. We constructed two tissue section arrays of major human organs (Figure 3A). The G97-A mAb indeed could detect neutrophils in normal tissues, such as colonic mucosa (Figure 3B). We also examined tissue cross-reactivity and found that mucous glands of the stomach and salivary glands displayed some non-specific staining (Figure 3A; Figure S2). Urothelial epithelium, Henle's loop, distal tubules, collecting ducts, and epididymis showed cytoplasmic granular staining. Importantly, these epithelial staining patterns were different from that seen in neutrophils (homogenous cytoplasmic/membranous staining). We further studied human inflammatory tissues, such as acute appendicitis and acute cholecystitis (Figure 3C). Neutrophils in appendix and gallbladder were strongly stained by the G97-A mAb. Finally, we investigated GPR97 expression patterns in several cancer tissues, such as high-grade gliomas, gastric adenocarcinomas, colon cancers, and colorectal liver metastasis. The G97-A mAb highlighted tumor-infiltrating neutrophils specifically and strongly in gastric cancer and colorectal liver metastasis (Figure 3D). On the other hand, reactive glial cells and glioma cells showed no immunoreaction with the G97-A mAb. We conclude that GPR97 is highly expressed by neutrophils in normal tissues as well as by infiltrating neutrophils in inflamed and certain cancer tissues.

### Inflammation enhances expression of GPR97

**Figure 4:**
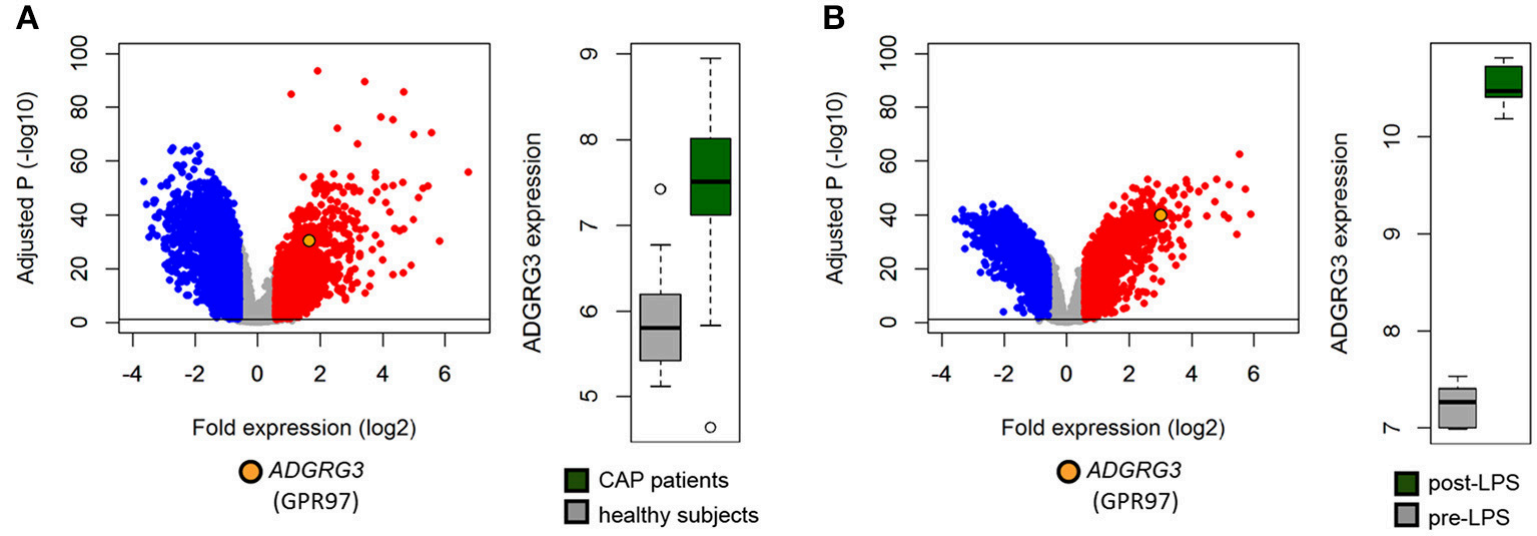
Inflammation enhances *ADGRG3* expression. Elevated whole blood expression of *GPR97* was detected in **(A)** critically ill community-acquired pneumonia patients (*n* = 101), relative to healthy subjects (*n* = 42), and **(B)** healthy volunteers at 4 h of endotoxemia (*n* = 7) initiated by administration 4 ng/kg *E. coli*-derived LPS, intravenously, relative to pre-LPS administration (*n* = 7). Red dots denote significant genes with fold expression >1.5. Blue dots denote significant genes with fold expression < -1.5. –log10 (BH) *p*, negative log-transformed Benjamini-Hochberg adjusted probabilities. Horizontal lines denote multiple comparison-adjusted probability of 0.05.

To determine whether neutrophils upregulate GPR97 under inflammatory conditions, we analyzed publicly available microarray data of blood gene expression from a cohort of community-acquired pneumonia patients (28, 29). *ADGRG3* was highly expressed in septic as compared to healthy subjects with fold expression equivalent to 3.2 (adjusted *p* = 4.3 × 10^−25^; Figure 4A). Furthermore, we analyzed publicly available microarray data of blood gene expression of healthy subjects administered 4 ng/kg LPS for 4 h relative to pre-LPS administration (30, 31). In this controlled clinical setting of acute systemic inflammation, *ADGRG3* expression was elevated at 4 h of human endotoxemia with fold expression equating to 4.7 (adjusted *p* = 4.4 × 10^−43^; Figure 4B). Our findings are in line with reports of enhanced *ADGRG3* transcription in PMNs of patients with severe trauma injury (39) as well as in blood cells of Parkinson's disease and type 2 diabetes patients (40).

### Mutation-induced activation of GPR97 suppresses cAMP production and increases NF-κB-mediated transcriptional activity

**Figure 5:**
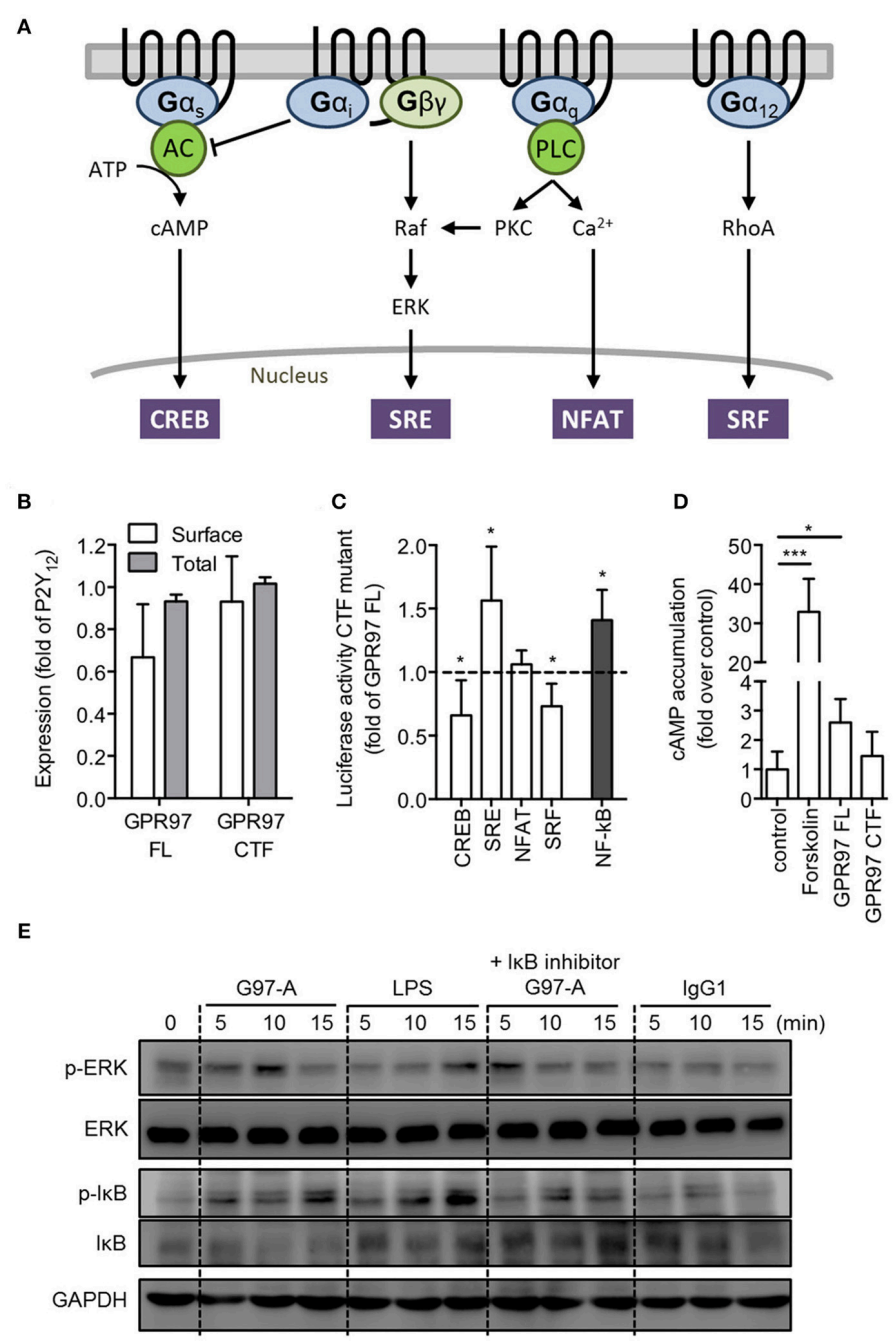
GPR97 mutation suppresses cAMP production and increases NF-κB activity enhances. **(A)** Schematic presentation of GPCR signaling pathways [adapted from Cheng et al. (41)]. Gα_s_-coupled receptors activate adenylate cyclase (AC), leading to cAMP accumulation. Gα_i_-coupled receptors inhibit AC, and their Gβγ subunits activate MAPK, such as ERK. Gα_q_-coupled receptors activate phospholipase C (PLC) to increase intracellular calcium concentration as well as activate protein kinase C (PKC), which results in Raf kinase activation of the MAPK pathway. Gα_12_-coupled receptors activate the small GTPase RhoA. Downstream reporters are cAMP response element-binding protein (CREB), serum response element (SRE), nuclear factor of activated T cells (NFAT), and serum response factor (SRF). **(B)** Surface and total cell expression of full-length (FL) and CTF-only mutant GPR97 in COS-7 cells. OD values are given as the percentage of human P2Y_12_, which served as a positive control (basal expression: empty vector: 0.01 ± 0.02 OD_492nm_; P2Y_12_: 1.304 ± 0.236 OD_492nm_). **(C)** Activity levels of GPR97–CTF in luciferase reporter gene assays on HEK-293T transfected cells. Provided is the percentage of the signal in relation to full-length GPR97 and after normalization to control in the given luciferase activity (full-length activity – fold over control: CREB: 1.31 ± 0.55; SRE: 1.12 ± 0.15; NFAT: 0.88 ± 0.22; SRF: 1.34 ± 0.57; NF-κB: 1.15 ± 0.17). **(D)** cAMP accumulation in COS-7 cells treated with forskolin or transfected with full-length and mutant GPR97. Data are shown as fold over control. **(E)** Isolated peripheral blood PMNs were incubated with control IgG1 or G97-A mAb (10 μg/ml) at 4°C, followed by a treatment with goat-anti mouse F(ab')2 at 4°C, and then shifted cells to 37°C for different time points. LPS treatment (100 ng/ml) served as positive control, and NF-κB signaling was inhibited with IκBα inhibitor (Bay 11–7082, 5 μM). Phospho- and total protein were detected by Western blot analysis. All data are means ± SEM of 4 independent experiments performed in triplicate. *p < 0.05; ***p < 0.001.

Luciferase reporter gene assay can be used to measure signaling of the four major G proteins (Gα_s_, Gα_i_, Gα_q_, and Gα_12_) by their downstream pathway (CREB, SRE, NFAT, and SRF; Figure 5A) (41). In order to analyze the intracellular signaling cascades that are governed by GPR97, we heterologously expressed full-length GPR97 and a truncated version of the receptor that lacks the N-terminal fragment (GPR97–CTF) in HEK-293T cells, as this type of mutation has been shown to induce constitutive activity of aGPCRs (42). Both receptor versions were sufficiently expressed (Figure 5B). In an initial screen, we tested full-length and CTF-only mutant GPR97 for the induction of transcription factor-mediated luciferase activity (Figure 5C). Compared to full-length GPR97, GPR97–CTF exhibited a significant increase in SRE (serum response element) and NF-κB (nuclear factor kappa-light-chain-enhancer of activated B cells) activity, while a significant reduction was detected in CREB (cAMP response element-binding protein) and SRF (serum response factor) activity, indicative of Gα_i_ coupling. However, direct cAMP measurements, showed a significant basal increase upon overexpression of full-length GPR97, which were markedly reduced through the CTF-only mutant (Figure 5D).

To complement the results of the *in vitro* signaling reporter assays in the heterologous cell over-expression system, we investigated the signaling events induced by GPR97 receptor ligation by incubating cold PMNs with G97-A mAb, followed by binding with goat-anti mouse F(ab')2, and then shifted cells to 37°C for different time points. Similar to LPS treatment, G97-A mAb initiated phosphorylation of IκBα (inhibitor of kappa B alpha) at 5 min. Faster than LPS treatment, G97-A mAb activated phosphorylation of the MAPK (mitogen-activated protein kinase) ERK (extracellular signal–regulated kinase) at 10 min. No such activation was observed when PMNs were incubated with control IgG1. Moreover, the phosphorylation of IκBα was inhibited in the presence of the IκB inhibitor Bay 11-7082, confirming the specific activation of NF-κB by GPR97 (Figure 5E). Interestingly, ERK activation was not affected by treatment with IκB inhibitor, suggesting that GPR97-mediated ERK is likely upstream or parallel of NF-κB signaling (Figure 5E). Taken together, we conclude that ligation and crosslinking of GPR97 receptor by the G97-A mAb induced intracellular signaling via the activation of ERK and NF-κB with a possible change in intracellular cAMP levels.

### Antibody ligation of GPR97 enhances antimicrobial mediator production of PMNs

**Figure 6:**
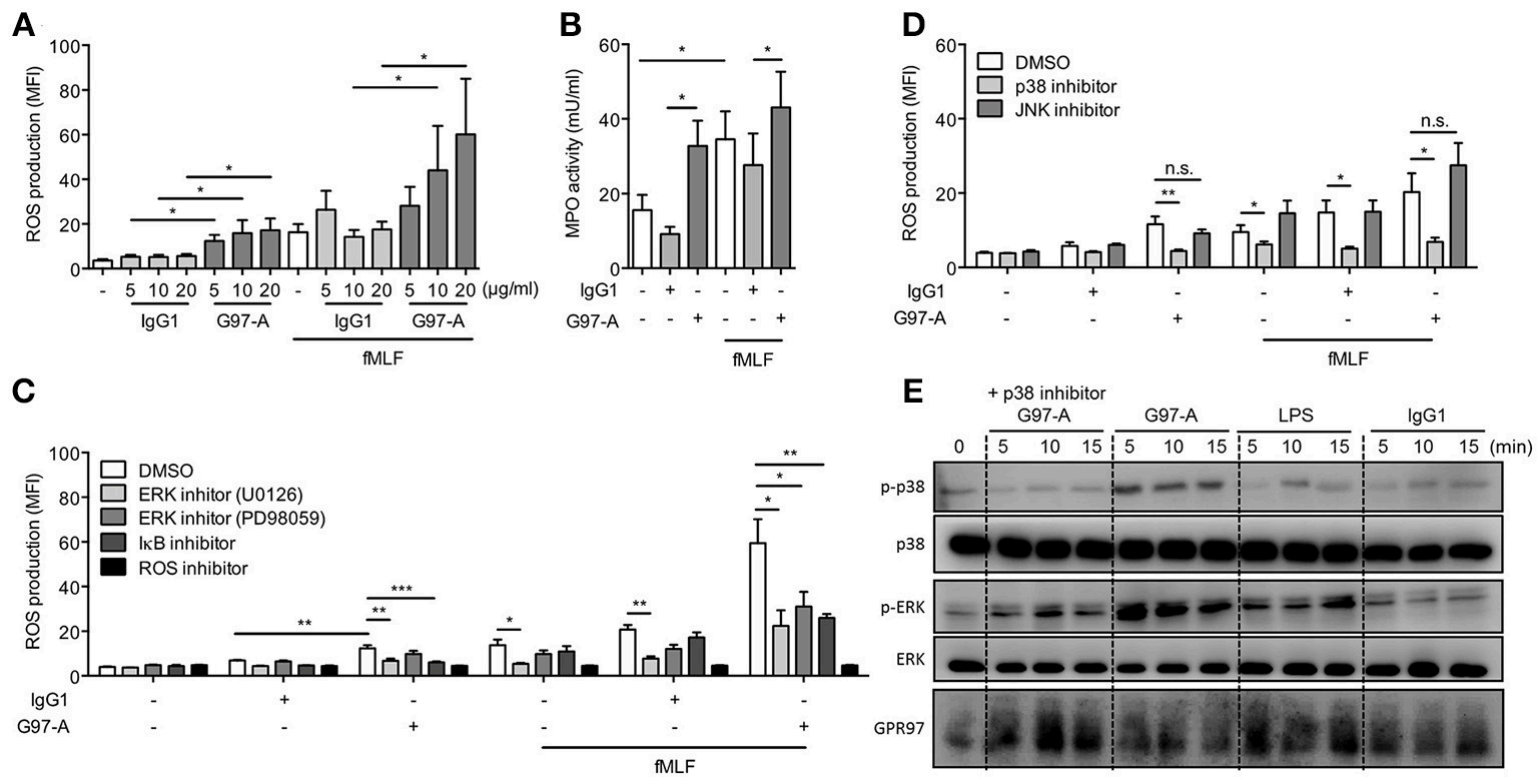
Antibody ligation of GPR97 enhances antimicrobial mediator production of PMNs. **(A,B)** Isolated peripheral blood PMNs, loaded with dihydrorhodamine 123, were respectively, incubated with indicated concentrations of immobilized control IgG1 or G97-A mAb (10 μg/ml) in the absence or presence of fMLF (1 μM). DMSO-treated cells were used a control. ROS production **(A)** and MPO activity **(B)** were measured by flow cytometry. Data are means ± SEM of 4 independent experiments. **(C**,**D)** Inhibitor pre-incubated PMNs were treated with immobilized control IgG1 or G97-A mAb (10 μg/ml) in the absence or presence of fMLF, and ROS production was measured. In **(C)**, NF-κB and ERK signaling as well as ROS activity were inhibited with IκBα inhibitor (Bay 11–7082, 5 μM), ERK inhibitors (U0126, 10 μM and PD98059, 20 μM), and ROS inhibitor (N-acetylcysteine, 10 mM), respectively. Data are means ± SEM of 4–7 independent experiments. In **(D)**, p38 and JNK signaling activities were inhibited with SB203580 (20 μM) and SP600125 (20 μM), respectively. Data are means ± SEM of 8 independent experiments. **(E)** Isolated PMNs were incubated with control IgG1 or G97-A mAb (10 μg/ml) at 4°C, followed by a treatment with goat-anti mouse F(ab')2 at 4°C, and then shifted cells to 37°C for different time points as indicated. LPS treatment (100 ng/ml) served as positive control, and p38 signaling was inhibited with SB203580 (20 μM). Phospho- and total protein were detected by Western blot analysis. MFI, mean fluorescence intensity; *p < 0.05; **p < 0.01; ***p < 0.001; ns, non-significant.

To examine the potential role of GPR97 in modulating PMN effector functions, PMNs were cultured on plates coated with G97-A mAb and treated with or without fMLF. Incubation with immobilized G97-A mAb alone modestly enhanced ROS production, similar to the effect fMLF alone. However, when PMNs were pre-incubated with the G97-A mAb, followed by fMLF treatment, the respiratory burst was augmented, suggesting an effective priming effect of G97-A mAb on PMNs (Figure 6A). The activating effect of G97-A mAb was only seen when the mAb was plate-immobilized; soluble G97-A mAb was ineffective (data not shown). In addition, PMNs treated with immobilized G97-A mAb had a higher MPO activity, comparable to that of fMLF-treated cells (Figure 6B). Of note, antibody ligation did not alter the morphology, adhesion, and apoptosis of neutrophils as well as GPR97 receptor internalization (Figures S3–S5).

Consistent with the signaling results, IκB inhibitor attenuated significantly the enhanced ROS production induced by G97-A mAb treatment alone, as well as by G97-A mAb plus fMLF (Figure 6C). By contrast, IκB inhibitor was not effective in down-regulating fMLF-induced ROS production, as seen in cells treated with fMLF alone or with fMLF plus control IgG1. As expected, the reaction was completely inhibited by the ROS inhibitor NAC. Interestingly, two widely used inhibitors of ERK signaling, U0126 and PD98059, showed a differential effect; U0126 displayed a significant inhibition of the enhanced ROS production induced by G97-A mAb or fMLF alone, as well as by G97-A mAb plus fMLF. By contrast, PD98059 seemed to be effective only in inhibiting cells treated with G97-A mAb plus fMLF (Figure 6C). As the two inhibitors work on the different cascade steps of ERK activation, they might have different inhibitory effects on GPR97-mediated signaling and its priming augmentation of fMLF-induced signaling. To examine the role of other MAPKs, signaling inhibitors of p38, and JNK were tested. Interestingly, p38 inhibitor but not JNK inhibitor also showed significant inhibition of the enhanced ROS production induced by G97-A mAb or fMLF alone, as well as by G97-A mAb plus fMLF (Figure 6D). This result indicated that ligation of GPR97 by G97-A mAb likely also induce p38 but not JNK signaling. Indeed, Western blot analysis showed that G97-A mAb induced p38 phosphorylation as early as 5 min and lasted until 15 min. Pre-treatment with p38 inhibitor completely inhibited the G97-A-induced phosphorylation of p38 (Figure 6E).

Beclomethasone dipropionate (BPD), a synthetic glucocorticoid with immunomodulatory properties, has been described to activate GPR97 through Gα_o_ (43). Interestingly, BPD itself did not affect the ROS production of PMNs but attenuated the ROS production induced by G97-A mAb (data not shown). However, BDP can also reduce fMLF-induced ROS production, and BDP is able to target the glucocorticoid receptor, so it is uncertain whether the inhibitory effect of BDP is indeed mediated via modulating GPR97 receptor signaling specifically. Taken together, these results further substantiate that ligation and activation of GPR97 by the immobilized G97-A mAb activates PMNs and modulates antimicrobial mediator production and enzymatic activity via the ERK/p38/NF-κB signaling pathways.

### Antibody ligation of GPR97 enhances bacteria uptake and killing of PMNs

**Figure 7:**
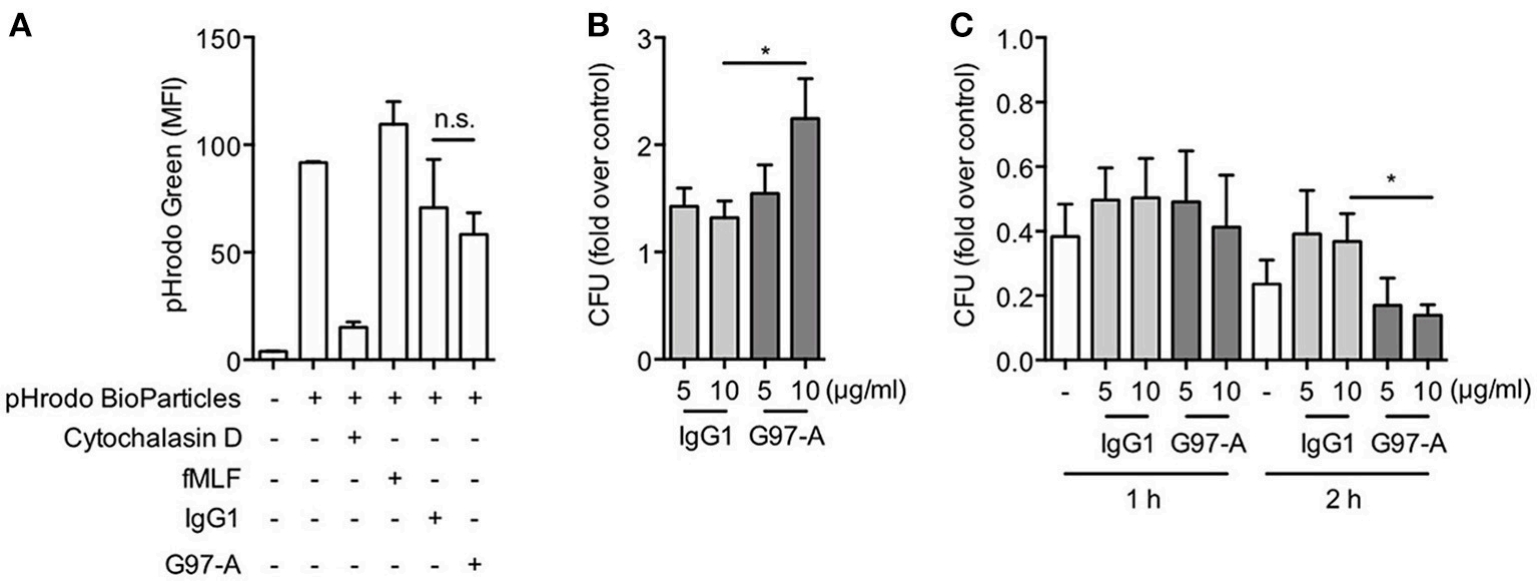
Antibody ligation of GPR97 enhances phagocytosis of live bacteria and bacteria killing activity of PMNs. **(A)** Phagocytosis of pHrodo™ Green *E. coli* BioParticles by PMNs treated with immobilized control IgG1 or G97-A mAb (10 μg/ml) was determined by flow cytometry. Data are means ± SEM of 3 independent experiments. MFI, mean fluorescence intensity. **(B)** Phagocytosis of live *E. coli* by PMNs treated with indicated concentrations of immobilized control IgG1 or G97-A mAb was determined by CFU counts. Data are means ± SEM of 8 independent experiments. **(C)** Bacteria killing activity of PMNs treated with indicated concentrations of immobilized control IgG1 or G97-A mAb for 1 h or 2 h determined by CFU counts. Data are shown as fold over control. Data are means ± SEM of 5 independent experiments. *p < 0.05; ns, non-significant.

Our results strongly suggested a role for GPR97 in the antimicrobial function of PMNs. To examine this potential function, phagocytosis, and bacteria killing assays were performed. PMNs were cultured on immobilized control IgG1 or G97-A mAbs, and their phagocytic uptake of pHrodo-labeled *E. coli* particles as well as live *E. coli* bacteria was measured. As shown, ligation of GPR97 by G97-A mAb did not increase the uptake of pHrodo-labeled *E. coli* particles but significantly enhanced the uptake of live *E. coli* at 10 μg/ml but not 5 μg/ml (Figures 7A,B). Likewise, the bacteria killing assays showed that G97-A mAb ligation significantly increased the bactericidal activity of PMNs at 10 μg/ml but not 5 μg/ml at 2 h but not 1 h incubation (Figure 7C). We conclude that GPR97 ligation enhances the uptake and killing of live *E. coli* by PMNs.

## Discussion

The aGPCR subfamily comprises 33 members in human, several of which are distinctly expressed and functionally involved in immune cells (4). We here describe the expressional, structural, functional, and signaling characteristics of GPR97. We show that *ADGRG3* transcription starts at the promyelocyte stage during granulopoiesis, occurs in all granulocyte lineages, and increases in acute and chronic inflamed and cancerous tissues, suggesting potential functional involvement, both in tissue homeostasis, inflammation, and tumorigenesis. Posttranslational modifications involve N-glycosylation and autocatalytic processing, resulting in a bipartite receptor that is detectable on the surface of circulating and tissue-infiltrating PMNs. Next to EMR1, EMR2, EMR3, and CD97, GPR97 is the fifth aGPCRs prominently expressed in PMNs (3, 4).

aGPCRs are able to activate G protein-dependent and -independent signaling pathways (4). In line herewith, previous studies have linked signaling molecules, including Gα_o_, NF-κB, and small GTPases, to GPR97 in different cell types (19, 24, 43). Several aGPCRs carry an encrypted tethered agonist within their ectodomain (44–46), termed the *Stachel* sequence, whose exposure has been suggested to be caused by either structural changes within the ECD or removal of the NTF (47). As aGPCRs have been shown to be responsive to mechanical stimuli (47–50), it is conceivable that these might play an essential role in the release of the *Stachel*. We tackled the issue of GPR97-mediated signaling first by applying a well-established *in vitro* signaling reporter assay in heterologous cells. We found that full-length GPR97 conducted Gα_s_/cAMP signaling, while a CTF-only mutant or crosslinking of the receptor triggered Gα_o/i_/ERK/NF-κB signaling. Direct analysis of GPR97-mediated signaling pathways in PMNs indeed revealed activation of NF-κB/IκBα as well as MAPKs ERK and p38. Most importantly, GPR97 ligation and activation led to the production and activation of antimicrobial mediators, including ROS and MPO. Specific inhibition of GPR97-induced ROS production by IκB and MAPK inhibitors further cemented the importance of these signaling molecules in GPR97-mediated cellular functions. Furthermore, bacteria uptake and killing are significantly enhanced by GPR97 ligation, confirming the antimicrobial functions of GPR97.

**Figure 8:**
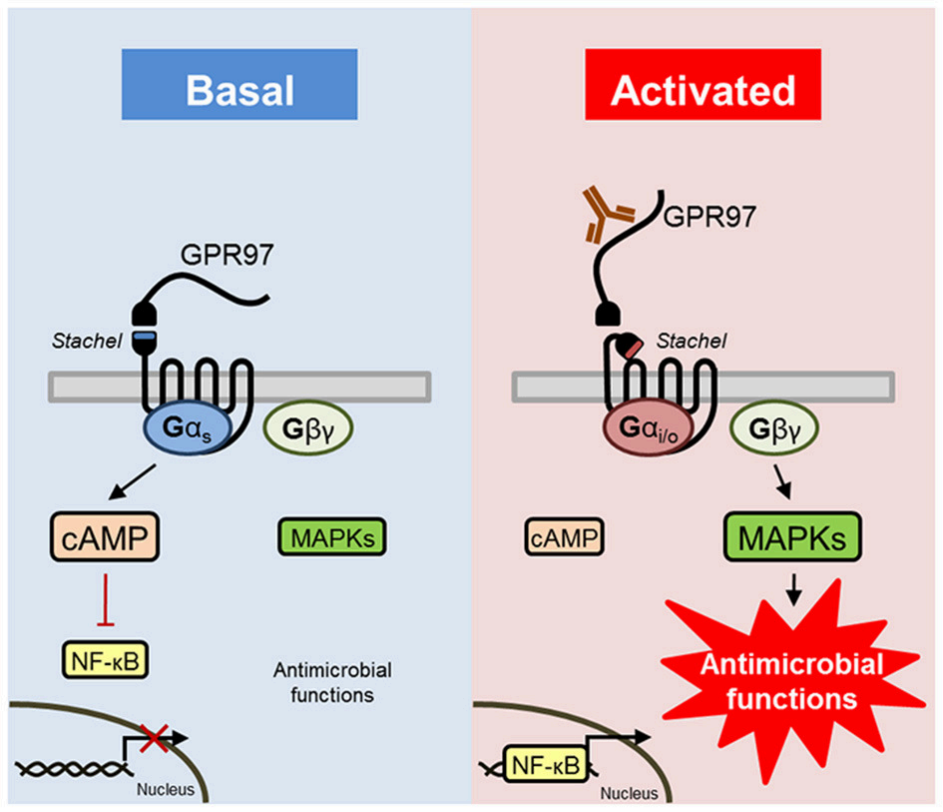
Schematic presentation of GPR97 signaling and function in human granulocytes. GPR97 signaling provides basal Gα_s_/cAMP-mediated signal transduction, which has been shown to inhibit NF-κB transcriptional activity (52). Removal or ligation of the NTF of GPR97 activates Gα_i/o_-induced reduction of cAMP levels, in combination with an increase in downstream effectors of Gβγ, such as NF-κB and MAPKs (ERK and p38), which triggers neutrophilic antimicrobial functions.

It was found that many GPCRs can interact with multiple G proteins. The binding selectivity of GPCRs for G proteins can be altered by additional factors, such as receptor oligomerization, conformational dynamics, basal activity, and ligand-induced changes (51). Hence, we hypothesized that GPR97 provides Gα_s_ signaling in the basal state, which has been shown to inhibit NF-κB transcriptional activity (52) (for a graphical summary, see Figure 8). In contrast, the active state of the receptor, induced through mechanical forces (crosslinking of receptor), structurally changes, or release of the NTF (GPR97–CTF), resulted in Gα_i_-protein signaling that then promoted NF-κB transcriptional activity and MAPK (ERK and p38) phosphorylation. In contrast to that, the global knock-out of GPR97 in mice results in increased CREB as well as augmented NF-κB p50/65 expression in primary splenocytes (19). It is however unclear whether this is a direct effect of GPR97 downstream signaling. We conclude that GPR97 balances signaling through controlled Gα_s_/Gα_i_-mediated cAMP/MAPK/NF-κB signaling, resulting in the regulation of effector functions in neutrophils.

MAPKs and NF-κB are well-established signaling molecules involved in inflammatory responses (53, 54). *ADGRG3*/GPR97 expression is enhanced in systemic inflammatory conditions [this study and (39, 40)]. The majority of GPCR signal transduction in neutrophils is mediated through the Gβγ subunits of Gα_i_ protein (55). Thus, the activation of MAPKs (ERK and p38) and NF-κB by Gα_i_ protein coupling is consistent with the pro-inflammatory role of GPR97 in neutrophils. Further strengthening a role of GPR97 as mediator of inflammation, a recent studied showed that GPR97 contributes to renal injury and inflammation in an acute kidney injury animal model by controlling the expression and activity of human antigen R (HuR), which is a RNA binding protein to regulate the stability of pro-inflammatory cytokine mRNAs (56, 57).

We conclude that GPR97 is a novel pan-granulocyte aGPCR in humans that triggers antimicrobial effector functions. The co-expression of multiple, distinct aGPCRs on PMNs prompts questions related to functional redundancy and receptor-specific activities. Indeed, EMR2 has also been shown to induce a priming effect of PMNs upon engagement by a mAb (15). More in-depth studies on these individual aGPCRs, including the identification of cognate ligands and detailed single-cell expressional profiles, should help in delineating these questions in the future.

## Author contributions

C-CH, T-YC, C-JW, MvdB, CP, BS, K-YI, IL, and T-CC performed experiments and interpreted results, C-CH, JHe, TK, BS, IL, JHa, and H-HL designed research and interpreted results, C-CH, IL, JHa, and H-HL wrote the paper.

### Conflict of interest statement

The authors declare that the research was conducted in the absence of any commercial or financial relationships that could be construed as a potential conflict of interest.

## Abbreviations

Ab: antibody
Ag: antigen
BPD: beclomethasone dipropionate
aGPCR: adhesion GPCR
cAMP: cyclic adenosine monophosphate
CFU: colony-forming unit
CTF: C-terminal fragment
CREB: cAMP response element-binding protein
ECD: extracellular domain
EMR: EGF-like module-containing mucin-like hormone receptor
ELISA: enzyme-linked immunosorbent assay
ERK: extracellular signal-regulated kinase
f-MLF: N-formyl-methionyl-leucyl-phenylalanine
GAIN: GPCR autoproteolysis-inducing
GPCR: G protein-coupled receptor
GPS: GPCR proteolysis site
HSPC: hematopoietic stem and precursor cell
IκB: inhibitor of kappa B
JNK: c-Jun N-terminal kinase
LPS: lipopolysaccharide
mAb: monoclonal antibody
MAPK: mitogen-activated protein kinase
MOI: multiplicity of infection
MPO: myeloperoxidase
NFAT: nuclear factor of activated T cells
NF-κB: nuclear factor kappa-light-chain-enhancer of activated B cells
NTF: N-terminal fragment
PMN: polymorphonuclear cell
ROS: reactive oxygen species
7TM: seven transmembrane
SRE: serum response element
SRF: serum response factor.
