# Supplementary material for: The Adhesion G Protein-Coupled Receptor GPR97/ADGRG3 Is Expressed in Human Granulocytes and Triggers Antimicrobial Effector Functions
Source: Front Immunol. 2018 Dec 3;9:2830. doi: 10.3389/fimmu.2018.02830 (PMC6287011; doi:10.3389/fimmu.2018.02830)
Supplement: Supplementary file 1 [file Data_Sheet_1.pdf]

Supplementary material

The adhesion G protein-coupled receptor GPR97/*ADGRG3* is expressed in human granulocytes and triggers antimicrobial effector functions

Cheng-Chih Hsiao[1,*], Tai-Ying Chu[2,*], Chia-Jung Wu[2], Maartje van den Biggelaar[3], Caroline Pabst[4], Josée Hébert[5,6], Taco W. Kuijpers[7,8], Brendon P. Scicluna[9,10], Kuan-Yu I[2], Tse-Ching Chen[11], Ines Liebscher[12], Jörg Hamann[1,#], and Hsi-Hsien Lin[2,11,13,#]

[1]Department of Experimental Immunology, Amsterdam UMC, University of Amsterdam, Amsterdam, The Netherlands; [2]Department of Microbiology and Immunology, College of Medicine, Chang Gung University, Taoyuan, Taiwan; [3]Department of Plasma Proteins, Sanquin Research and Landsteiner Laboratory, Amsterdam UMC, University of Amsterdam, Amsterdam, The Netherlands; [4]Department of Medicine V, Heidelberg University, Heidelberg, Germany; [5]Division of Hematology-Oncology and Leukemia Cell Bank of Quebec, Maisonneuve-Rosemont Hospital, Montreal, QC, Canada; [6]Department of Medicine, University of Montreal, Montreal, QC, Canada; [7]Department of Blood Cell Research, Sanquin Research and Landsteiner Laboratory, Amsterdam UMC, University of Amsterdam, Amsterdam, The Netherlands; [8]Department of Pediatric Hematology, Immunology and Infectious Diseases, Emma Children's Hospital, Amsterdam UMC, Amsterdam, The Netherlands; [9]Department of Clinical Epidemiology, Amsterdam UMC, University of Amsterdam, Amsterdam, The Netherlands; [10]Center for Experimental Molecular Medicine, Amsterdam UMC, University of Amsterdam, Amsterdam, The Netherlands; [11]Department of Anatomic Pathology, Chang Gung Memorial Hospital-Linkou, Taoyuan, Taiwan; [12]Rudolf Schönheimer Institute of Biochemistry, Medical Faculty, Leipzig University, Leipzig, Germany, and [13]Chang Gung Immunology Consortium, Chang Gung Memorial Hospital-Linkou, Taoyuan, Taiwan; [*, #]Equal contribution

SUPPLEMENTARY MATERIALS AND METHODS

Antibody neutralization assay

GFP-positive HEK-293T cells transfected with pReceiver–GPR97–GFP (GeneCopoeia, MD, USA) were sorted and used for subsequent analysis. Cells were blocked for 1 h in cold blocking buffer (1% BSA, 5% normal goat serum in PBS), followed by incubation with GPR97-A mAb (5 μg/ml) with or without mFc or GPR97–mFc (20 μg/ml) at 4°C for 1 h. Cells were then washed extensively and incubated for 1 h with Alexa Fluor 647-conjugated goat anti-mouse IgG (2 μg/ml; Thermo Fisher) in blocking buffer. Cells were washed again extensively and subjected to analysis by flow cytometry.

Adhesion assay

Isolated PMNs (1×10^6 cells/ml) were suspended in RPMI and incubated with immobilized control IgG1, G97-A mAb (10 μg/ml), or PMA (2 nM; Sigma-Aldrich) at 37°C for 1 h. Following gentle washing with PBS, cells were incubated with WST-1 (Roche) at 37°C incubator for 30 min. The levels of adherent cells were measured by the absorbance at OD_{440}.

Apoptosis assay

Isolated PMNs (2×10^6 cells/ml) suspended in RPMI were incubated with immobilized control IgG1, G97-A mAb (10 μg/ml), or LPS (200 ng/ml) for 20 h. Cell apoptosis was analyzed using the Annexin V-FITC Apoptosis Kit (Biovision, Milpitas, CA, USA) and flow cytometry.

Receptor internalization assay

Isolated PMNs were suspended in ice-cold PBS buffer supplemented with 1% BSA and 5% normal goat serum. Cells were then incubated with Alexa Fluor 647-labeled G97-A mAb (Invitrogen) at 4°C for 1 h. Following extensive washing, cell samples were transferred to 37°C for 10 min, 30 min, and 60 min, respectively. When necessary, cells were treated with dynasore (50 μM; Sigma-Aldrich) or cytochalasin D (1 μM; Sigma-Aldrich) to stop receptor internalization. The level of receptor internalization was detected by flow cytometry.

FIGURES

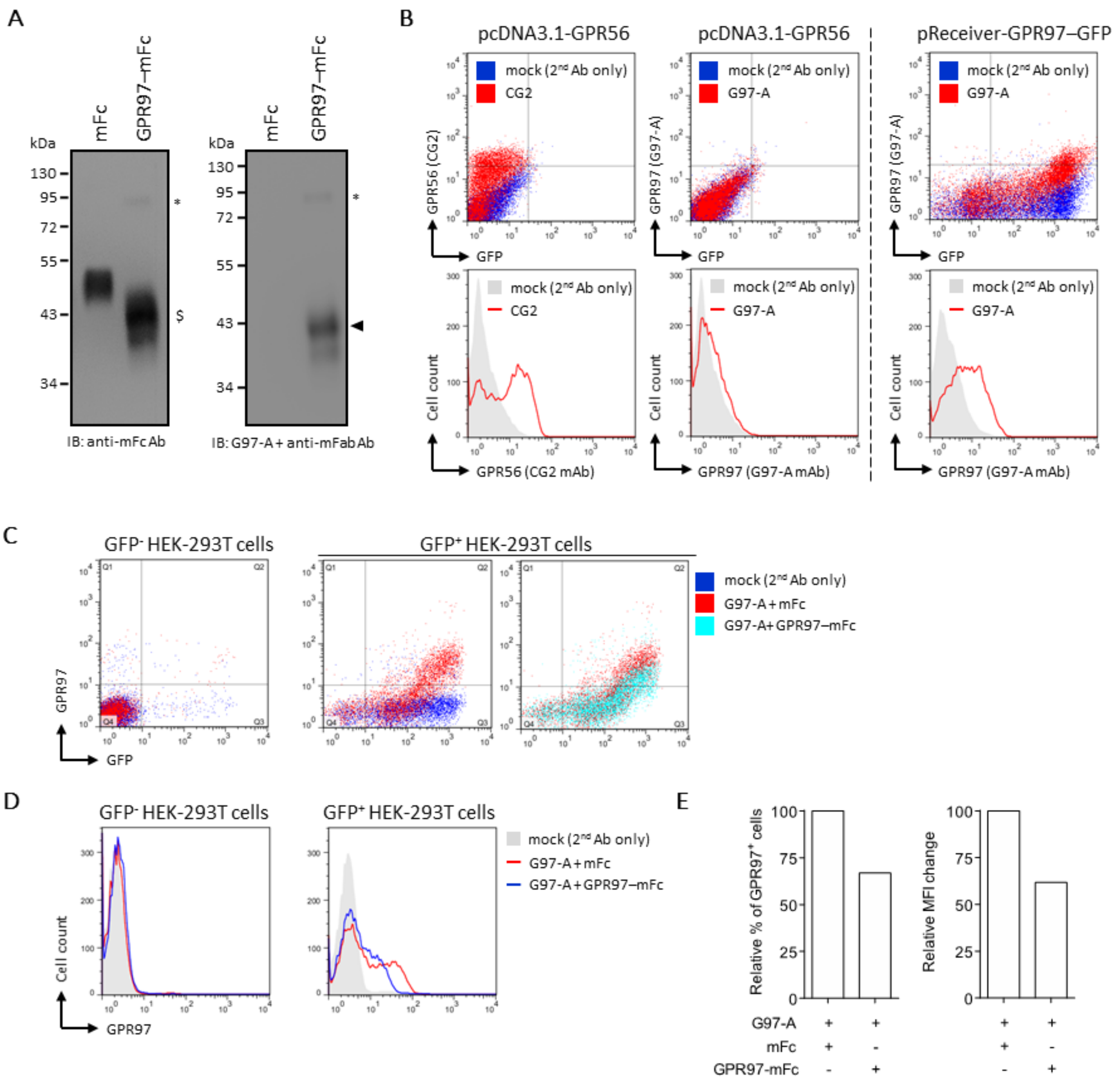

Figure S1. Determination of the specificity of G97-A mAb. (A) Western blot analysis of the GPR97-ECD–mFc protein using Abs specific to mFc (left panel) and GPR97-ECD (right panel). For the detection of mFc (left panel), anti-mouse IgG (Fc specific)-peroxidase was used, whereas the GPR97-ECD (right panel) was detected using G97-A as first Ab, followed by anti-mouse IgG (Fab specific)-peroxidase. The molecular weight of GPR97-NTF is ~43 kDa (indicated by arrowhead), very similar to the size of mFc fragment (indicated by the dollar sign). A minor fraction of uncleaved GPR97-ECD–mFc was expressed as a ~80-kDa band and detected by both Abs (indicated by asterisk). The soluble mFc was included as a control and appeared slightly larger than the cleaved mFc fragment of the GPR97-ECD–mFc protein due

to additional sequences encoded by pSecTag2A-mFc expressional construct, which contain multiple cloning sites and an additional biotinylation sequence. (**B**) Flow-cytometric analysis of HEK-293T cells transfected with pcDNA3.1-GPR56 and pReceiver-GPR97–GFP with mAb directed against GPR56 (CG2) and GPR97 (G97-A). (**C**) Flow-cytometric analysis of HEK-293T cells transfected with pReceiver-GPR97–GFP. GFP^{-} and GFP^{+} cell populations were sorted by flow cytometry and stained for surface GPR97 expression using G97-A mAb. To demonstrate specificity, G97-A mAb was pre-incubated with a 2-fold concentration of soluble mFc or GPR97–mFc before flow cytometry. (**D**) Flow-cytometric analysis of GPR97 surface levels in sorted GFP^{-} and GFP^{+} GPR97-expressing HEK-293T cells using G97-A mAb. Cells were pre-incubated with a 2-fold concentration of soluble mFc or GPR97–mFc before flow cytometry. (**E**) Quantification of the flow cytometry plots shown in panel D.

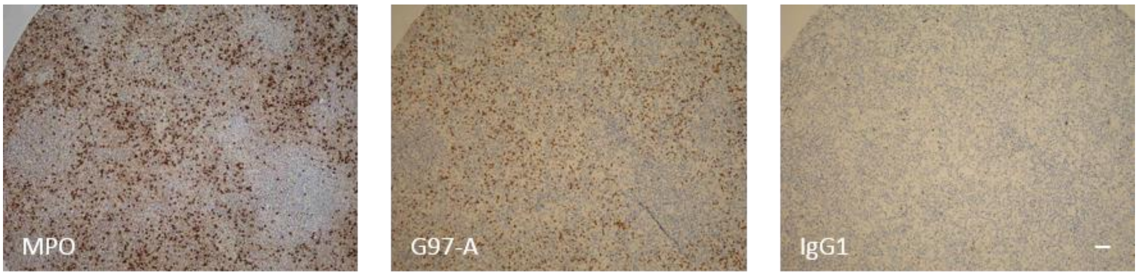

Figure S2. Specific GPR97 expression in tissue-infiltrating PMNs. The specific reactivity of the G97-A mAb on tissue-infiltrating neutrophils was examined on formalin-fixed and paraffin-embedded human spleen tissue sections by comparing the staining patterns obtained with anti-MPO, G97-A, and control IgG1 mAb as indicated. Scale bar: 50 µm.

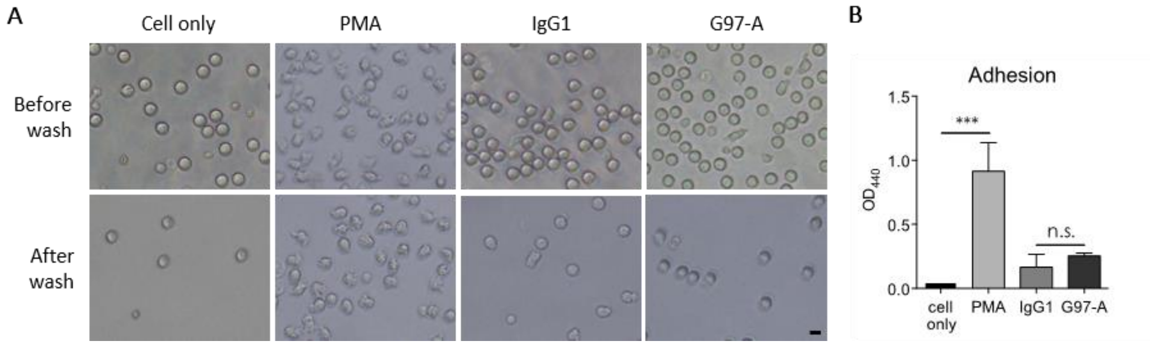

Figure S3. GPR97 activation does not alter the morphology and adhesion of neutrophils. (**A**) Purified human neutrophils were cultured in the absence or presence of PMA (2 nM) or on plates pre-coated with control IgG1 or G97-A mAb (10 µg/ml) for 1 h before gentle washing and microscopic analysis of cell adhesion. Cell morphology of neutrophils before and after washing is shown. Scale bar: 10 µm. (**B**) Quantitative comparison of cells adhesion at the indicated conditions. Data are means ± SEM of 3 independent experiments. *** p <0.001; ns, non-significant.

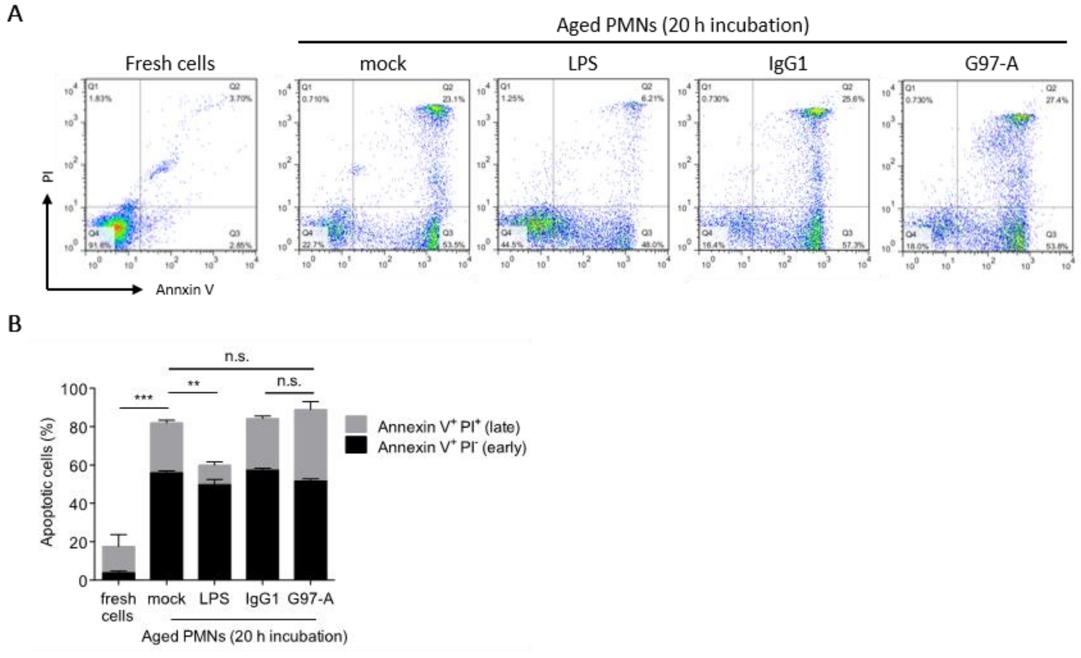

Figure S4. GPR97 activation does not affect neutrophil apoptosis. (**A**) Cell apoptosis of fresh and aged PMNs (cultured at 37°C incubator for 20 h) was detected using flow-cytometric analysis of cells stained with Annexin V and propidium iodide (PI). (**B**) Quantitative comparison of cells apoptosis at the indicated conditions. Data are means ± SEM of 3 independent experiments. ** p <0.05; *** p <0.001; ns, non-significant.

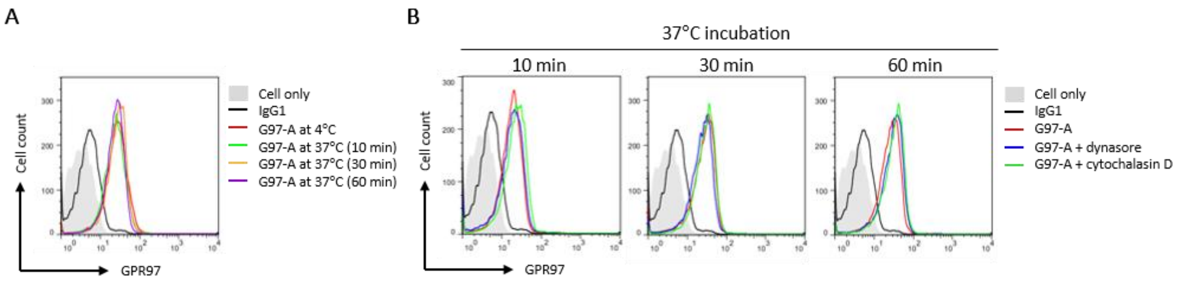

Figure S5. GPR97 mAb binding does not induce GPR97 receptor internalization. (**A**) Flow cytometric analysis of freshly isolated PMNs stained by Alexa Fluor 647-labeled control IgG1 or G97-A mAb (5 µg/ml) and incubated at 4°C or shifted from 4°C to 37°C for 10, 30, and 60 min as indicated. (**B**) PMNs were treated without or with dynasore (50 µM) or cytochalasin D (1 µM) before staining with Alexa Fluor 647-labeled G97-A mAb (5 µg/ml). Cells were stained at 4°C then shifted to 37°C. Cell surface GPR97 levels were measured by flow cytometry at different time points as indicated.
